# Supplementary material for: Clinical Usefulness of FRAX Score for Predicting Sarcopenia in Patients with Chronic Liver Disease
Source: J Clin Med. 2021 Sep 9;10(18):4080. doi: 10.3390/jcm10184080 (PMC8465236; doi:10.3390/jcm10184080)
Supplement: Supplementary file 1 [file jcm-10-04080-s001.zip › jcm-1323139-supplementary.pdf]

Table S1. The 10-year probabilities of major osteoporotic and hip fractures based on the FRAX with BMD.

10-year probability of fracture	ALL patients	BMD (+) high risk	BMD (+) non-high risk
Major osteoporotic fracture (%)	9.3 (5.2–16.0)	18.0 (13.0–27.3)	5.7 (4.1–8.1)
Hip fracture (%)	1.9 (0.7–5.3)	6.1 (4.4–11.3)	0.8 (0.3–1.7)

BMD, bone mineral density.

Table S2. The 10-year probabilities of major osteoporotic and hip fractures based on the FRAX without BMD.

10-year probability of fracture	ALL patients	BMD (–) high risk	BMD (–) non-high risk
Major osteoporotic fracture (%)	14.0 (7.4–22.0)	21.0 (15.0–31.0)	6.8 (5.1–8.2)
Hip fracture (%)	4.6 (1.3–11.0)	9.5 (5.8–16.0)	1.1 (0.6–1.7)

BMD, bone mineral density.

Table S3. Univariate analysis for factors associated with sarcopenia.

Variable	OR (95% CI)	*p*-value
Gender (Men)	0.904 (0.547–1.494)	0.694
Age (years)	1.095 (1.060–1.131)	< 0.001
BMI (kg/m²)	0.738 (0.671–0.812)	< 0.001
Liver cirrhosis	1.933 (1.165–3.205)	0.011
Glucocorticoid use	1.539 (0.593–3.998)	0.376
Smoking	0.864 (0.479–1.557)	0.626
Alcohol intake	0.877 (0.379–2.027)	0.759
Etiology	0.909 (0.724–1.142)	0.414
Total bilirubin (mg/dL)	1.189 (0.811–1.744)	0.375
Albumin (g/dL)	0.421 (0.255–0.696)	< 0.001
Prothrombin time (%)	0.991 (0.975–1.007)	0.247
IGF-1 (ng/mL)	0.975 (0.965–0.985)	< 0.001
BCAA (μmol/L)	0.991 (0.987–0.994)	< 0.001
Osteoporosis	6.319 (3.684–10.838)	< 0.001
Prevalent fracture	3.791 (2.251–6.386)	< 0.001
High fracture risk (FRAX with BMD)	6.655 (3.803–11.644)	< 0.001
High fracture risk (FRAX without BMD)	5.264 (2.766–10.017)	< 0.001

BCAA, branched-chain amino acid; BMD, bone mineral density; BMI, body mass index; CI, confidence interval; FRAX, Fracture Risk Assessment tool; IGF-1, insulin-like growth factor 1; OR, odds ratio.

Table S4. Significant factors associated with sarcopenia in patients with chronic liver disease.

	Univariate		Multivariate	
Variable	OR (95%CI)	*p*-value	OR (95%CI)	*p*-value
Age (years)	1.095 (1.060–1.131)	< 0.001	1.055 (1.014–1.097)	0.008
BMI (kg/m²)	0.738 (0.671–0.812)	< 0.001	0.761 (0.681–0.852)	< 0.001
Liver cirrhosis	1.933 (1.165–3.205)	0.011		
Albumin (g/dL)	0.421 (0.255–0.696)	< 0.001		
IGF-1 (ng/mL)	0.975 (0.965–0.985)	< 0.001	0.980 (0.967–0.993)	0.003
BCAA (μmol/L)	0.991 (0.987–0.994)	< 0.001	0.995 (0.991–0.999)	0.007
Osteoporosis	6.319 (3.684–10.838)	< 0.001		
Prevalent fracture	3.791 (2.251–6.386)	< 0.001		
High fracture risk*	6.655 (3.803–11.644)	< 0.001	3.143 (1.559–6.340)	0.001

BCAA, branched-chain amino acid; BMI, body mass index; CI, confidence interval; IGF-1, insulin-like growth factor 1; OR, odds ratio. *High fracture risk based on the Fracture Risk Assessment tool with bone mineral density.

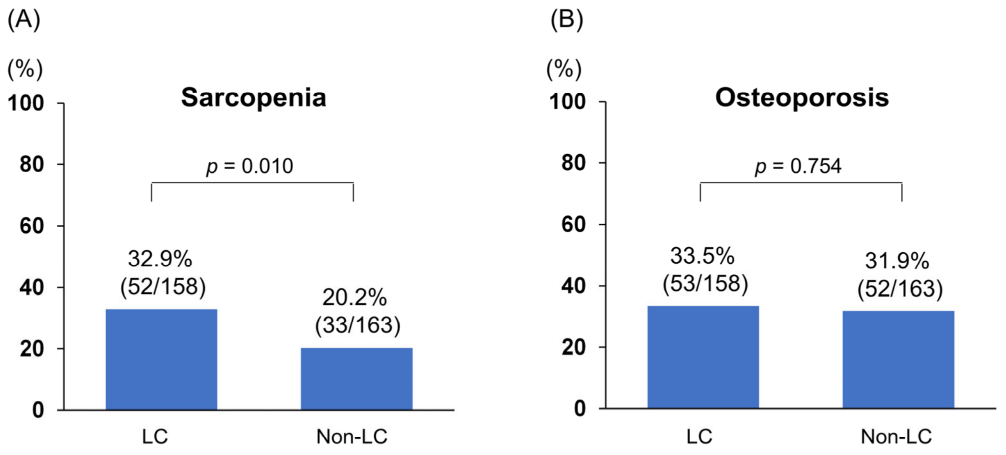

Figure S1. The prevalence of sarcopenia and osteoporosis between patients with and without liver cirrhosis (LC). (**A**) The prevalence of sarcopenia was significantly higher in the LC group than in the non-LC group ($p = 0.001$). (**B**) The prevalence of osteoporosis was not significantly different between the two groups.
